# Investigating the stretch-shortening cycle fatigue response to a high-intensity stressful phase of training in collegiate men's basketball

**DOI:** 10.3389/fspor.2024.1377528

**Published:** 2024-04-18

**Authors:** Nicolas M. Philipp, Ramsey M. Nijem, Dimitrije Cabarkapa, Charles M. Hollwedel, Andrew C. Fry

**Affiliations:** Jayhawk Athletic Performance Laboratory – Wu Tsai Human Performance Alliance, University of Kansas, Lawrence, KS, United States

**Keywords:** sport science, neuromuscular fatigue, force plate, athlete monitoring, countermovement jump, repeat hop test

## Abstract

**Introduction:**

While using force-plate derived measures of vertical jump performance, reflective of stretch-shortening-cycle (SSC) efficiency is common practice in sport science, there is limited evidence as to which tests and measures may be most sensitive toward neuromuscular fatigue. The aim of this study was to explore the SSC fatigue response to a one-week high-intensity fatiguing phase of training in National Collegiate Athletic Association (NCAA) Division-I basketball players.

**Methods:**

The study timeline consisted of three weeks of baseline measures, one week of high-intensity training, and two weeks of follow-up testing. Countermovement jumps (CMJ) and 10-5 hop tests were performed at baseline, as well as at two time-points during, and three time-points following the fatiguing training period, allowing for performance-comparisons with baseline.

**Results:**

Compared to the weekly training sum at baseline, during the high intensity training phase, athletes were exposed to very large increases in selected external load metrics (ES = 1.44–3.16), suggesting that athletes experienced fatigue acutely, as well as potential longer lasting reductions in performance. Vertical jump data suggested that in the CMJ, traditional metrics such as jump height, as well as metrics reflecting kinetic outputs and movement strategies, were sensitive to the stark increase in high-intensity training exposure. The 10-5 hop test suggested a fatigue-induced loss of tolerance to ground impact reflected by performance reductions in metrics related to jump height and reactive strength qualities.

**Discussion:**

These findings emphasize that when monitoring neuromuscular fatigue, variables and assessments may not be looked at individually, but rather as part of a more global monitoring approach.

## 1 Introduction

The management of neuromuscular fatigue (NMF) in athletic populations has been of interest to scientists and practitioners for a considerable amount of time, with implications on health and performance (1–5). Several different definitions for neuromuscular fatigue (NMF) have historically been used. According to Cairnes et al. the phenomenon of neuromuscular fatigue in humans is described readily in subjective terms and is measured objectively as an acute reduction in performance during exercise; however, the underlying physiological mechanisms are controversial and are the focus of a considerable amount of research (6). Others have defined neuromuscular fatigue as an exercise-induced reduction in maximal voluntary force (7), or the inability to produce a certain expected force or power (8). Combined, neuromuscular fatigue may be thought of as a reduction in the maximal voluntary force induced by exercise, with neuromuscular function changes that are due to repeated or sustained muscular contraction, and that are produced either at the peripheral or central levels, and that can be detected acutely, and for upwards of 48 h to an extended period (1). Basketball in particular is a sport in which dense training and competition schedules are a common challenge to navigate for sport coaches and support staff, with loads varying based on the time of year, and fatigue identification and mitigation being one of the tasks of the modern sport scientist. Therefore, practitioners working in such environments must be equipped with the analysis tools needed to quantify, analyze, and understand factors related to NMF.

Not necessarily a very recent evolution, the stretch-shortening cycle (SSC) has been proposed as a powerful model to study naturally occurring NMF in humans (9). Consisting of an eccentric action, where the preactivated muscle is first lengthened, followed by a concentric muscle action during which stored elastic energy is harnessed to enhance the force generation, the SSC presents itself as a more wholistic and natural concept of studying NMF, compared to isolated forms such as purely eccentric, isometric, or concentric actions (9, 10). Taking into account that ground contact phases of running, jumping, and hopping all utilize the SSC, it seems reasonable to suggest that these actions themselves (e.g., countermovement vertical jump, drop jump, hop test), as well as repeated or continuous bouts of moderate to high-intensity SSC-type activities, may be used to study the neuromuscular fatigue phenomenon from a dose and a response standpoint (9–11). According to Nicol et al. when SSC exercise is performed repeatedly with high intensity, over a long duration, reversible neural, structural, and mechanical disturbances may be seen, with severity and duration being dependent on the nature of the SSC task (9).

In most cases using the SSC, different vertical jump tasks have been identified as common means to study neuromuscular function within high-performance sports (12). When using force plates, more detailed insights may be gleaned from tasks such as countermovement jumps or drop- and repeated hop jump tests, such as impulse and peak and average force measures, in addition to power performance parameters, including temporal values across different phases (e.g., braking vs. propulsive). Particularly in sports such as basketball in which irregular and fairly dense schedules are a common theme, force plates have become a central tool for studying neuromuscular performance as it relates to screening, profiling, monitoring, and rehabilitating athletes (13–15). However, even though such procedures have become a standard across amateur and professional sports organizations, there still seems to be limited scientific consensus regarding which force-plate-derived metrics may be most useful or sensitive, especially when studying constructs such as NMF. For instance, a recent systematic review looking into the kinetic and kinematic aspects of the vertical jump related to overreaching has determined that metrics such as peak power, peak force, and jump height demonstrated the most consistent negative alterations (16). What must be acknowledged however, is that all three of those metrics tend to display large degrees of inter-correlation, and that a vast number of other force-time characteristics may be gleaned from vertical jump tasks, when performed on force plates (17, 18). Another very recent commentary on helping practitioners select countermovement vertical jump (CMJ) metrics that matter with regards to performance profiling, NMF monitoring, and rehabilitation, suggested time-, or strategy-based metrics such as time-to-takeoff or propulsive phase duration, in addition to reactive strength index-modified (mRSI; i.e., ratio of jump height and time-to-takeoff) to be most sensitive to or correspond with neuromuscular fatigue (19). These suggestions are primarily based on work by Gathercole et al. who studied the response of 8 college-level team sport athletes to a 3-stage Yo-Yo fatiguing protocol, using traditional and alternative, CMJ-derived force-time metrics (20). Primary findings indicate that at 24 h post-fatigue protocol, most metrics trended back toward baseline, with small increases in time-dependent variables (e.g., eccentric and concentric duration) observed at 72 h post-fatigue protocol (20). Within a different study on a small cohort of Olympic-level snowboard athletes, the same group of authors proposed that acute lower-body fatigue exercise protocol was capable of inducing a notable decrease in force production and an increase in jump duration (20). Conversely, in response to training, a chronic adaption was an increase in force production and a decrease in CMJ durations (21). Combined, these two studies suggest that alternative force-time metrics displaying the athlete's jump strategy may be more sensitive to NMF when compared to outcome-based metrics such as jump height. Similarly, in response to NMF through training and game performance in female basketball players, Spiteri et al. proposed that jump height remained relatively unchanged, while jump duration increased, highlighting that flight-time:contraction-time may be a metric suited to study fatigue (22). On the other hand, others have proposed jump height to be sensitive to acute levels of fatigue (23, 24). Further, Spencer et al. recently aimed to explain maintenance in jump height following soccer competition through decreases in jump momentum, induced by acute reductions in body weight, which should be factored in when interpreting the acute fatigue response using vertical jump tasks (25). Combined, the previous research reports (20–25) reveal the complexity of making sense of which measures may be most sensitive toward NMF.

While not applicable to all of the aforementioned studies, some limitations should be acknowledged. In many cases, researchers implemented a limited amount (in some cases only one) post-fatigue test session to study the trajectory of the response (16, 25). Previous research has highlighted that SSC often presents itself in a bimodal fashion with immediate performance reductions induced by metabolic fatigue, and later reductions in neural factors through structural damage (e.g., muscle soreness), which could lead to changes in mechanical parameters (9). In some cases, full recovery may take 4–8 days, depending on the parameter and severity of the fatigue stimulus (9). Therefore, only implementing one immediate post-fatigue follow-up, or stopping follow-ups at 24-, or 72-h post-fatigue, likely fails to paint a complete picture of the athletes' NMF response. Beyond that, the modality used to induce fatigue deserves some attention as well. While some have aimed to study a NMF response to sport-specific tasks such as practices or games, although often failing to report demands (e.g., external load exposure), others have implemented fatigue protocols, which outside of the study procedures may rarely be experienced by athletes. Further, such protocols are often implemented at a single time point, rather than over multiple days, mimicking schedules seen in athletic settings.

In line with said limitations and in an effort to provide sport science practitioners with additional evidence about the sensitivity of force-time metrics derived from SSC tasks, the aim of this study was to explore the SSC fatigue response to a one-week pre-season high-intensity, stressful phase of training in National Collegiate Athletic Association (NCAA) Division-I basketball players.

## 2 Materials and methods

### 2.1 Experimental design

Our study timeline consisted of a three-week baseline period, during which weekly baseline measurements for the dependent neuromuscular performance variables, in addition to external practice loads were gathered. During each baseline week, athletes performed 3 CMJ trials, and two 10-5 hop test trials on separate days. The days and times at which these tests were performed remained constant over the baseline period (e.g., 14:00–16:00 h). During and following the fatiguing training period, CMJs and 10-5 hop tests were performed together on the same day. The CMJ and the 10-5 hop test were chosen to represent a slow and a fast SSC movement, respectively (26). The third baseline week was used to gather external load on athletes, to establish what all aspects of a usual training week looks like from a load perspective. A 5-day high-intensity stressful training phase was used as the SSC fatigue stimulus, and measures on dependent variables were collected at numerous time points preceding, during, and following the stressful training phase. All three baseline weeks as well as the fatiguing 5-day high-intensity training period were part of the NCAA 8-h preseason period, which allows for 8 h of organized on-court and strength and conditioning activities, in addition to film sessions and team demands. During this period, athletes only participated in training, and no games were played.

**Figure 1:**
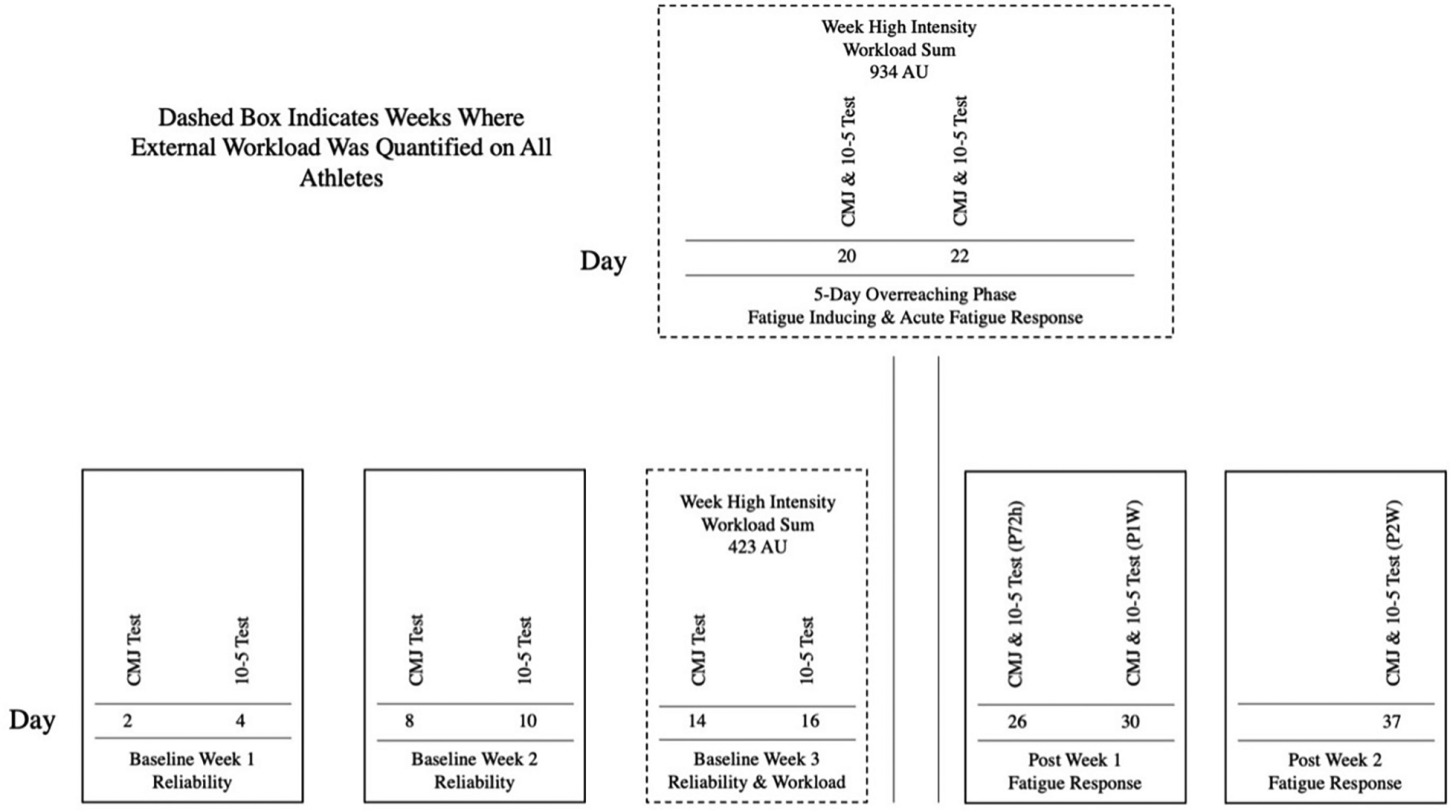
Schematic representation of the study timeline. CMJ, countermovement jump.

The two weeks following the fatiguing 5-day high-intensity training period were part of the NCAA 20-h period, which allows for 20 h of organized team practices and strength and conditioning sessions in addition to other team demands. The stressful training phase consisted of five consecutive days during which athletes participated in basketball-specific, high-intensity SSC-dependent drills such as repeated line sprints, repeated vertical jump tasks, as well as repeated lateral shuffle and change of direction drills. All training sessions during the stressful training phase lasted for about 60 min. In addition to these five sessions, which did not include basketball practice, athletes additionally participated in one organized basketball practice. Further, two strength and conditioning sessions were implemented during this week, which were primarily used to gather study-related data and for recovery purposes (i.e., static and dynamic mobility work). During the fatiguing 5-day high-intensity training phase, CMJs and 10-5 hop tests were performed on two days (both tests performed on the same day), 6 h following the training session (A1 and A2). Following the fatiguing training phase, further CMJ and 10-5 hop tests were performed at 72 h post (P72h), as well as 1-, and 2-weeks post (P1W and P2W). External load data was only collected during the third baseline week, as well as during the fatiguing 5-day high-intensity training phase. More detail on respective procedures is provided below. Figure 1 shows a schematic of the study timeline.

### 2.2 Subjects

Authors conducted a power analysis using G*Power, with an effect size *f* = 0.25, a significance level of 0.05, a desired power level of 0.95, one group, six measurements per participant, and a correlation of 0.75 among repeated measures. The analysis suggested that a total sample size of 15 participants would be needed to achieve the desired level of statistical power. Sixteen male, college-age (18–25 years) basketball players, currently on an active roster at an NCAA Division-I university participated in this study (body mass = 93.2 ± 11.3 kg, height = 195.6 ± 10.4 cm). All subjects were healthy and cleared by the university's sports medicine staff throughout the duration of the study. Outside of the three-week baseline period, which was implemented to gather reliability data, all athletes presented with sufficient experience in performing both the CMJ and 10-5 hop test. Out of 16 athletes, ten had at least two years of previous experience performing CMJs on a near weekly basis, while 6 out of 10 athletes had at least four months of previous experience, with performing CMJs on a near weekly basis. Further, all 16 athletes had sufficient exposure to the 10-5 hop test throughout the 4 months leading up to the start of the study. Lastly, all athletes provided their written consent for their deidentified data to be used for research purposes, as approved by the Universities Institutional Review Board. This study was conducted in accordance with the Declaration of Helsinki.

### 2.3 Countermovement jump testing

Procedures to collect CMJ data as a test reflecting the use of a slow SSC were adapted from previous studies (27, 28). On test days athletes performed three CMJ's with their hands placed on their hips, with individual jumps being separated by 15–30 s. Jumps were performed at the beginning of a weight room-based training session, following a static and dynamic warmup. Ground reaction force data was collected via two sets of dual, unidimensional force plates, sampling at 1,000 Hz (Hawkin Dynamics, Westbrook, ME, USA), and force plates were zeroed/calibrated prior to each data collection. Athletes were instructed to step onto the force plate and to stand still with their hands on the hips until a sound accompanied by a flash on a tripod-mounted tablet directly in front of them was given. Following this, they were asked to jump as fast and as high as possible, while keeping their hands on the hips. Strong verbal encouragement was provided during the entire movement, to ensure that maximal effort was given during each jump. Following manufacturer guidelines, individual CMJs were divided into subphases consisting of unweighting, braking, propulsive, flight, and landing phase (29). Metrics of interest were primarily selected from the braking and propulsive phase and were based on the authors' hypotheses about fatigue-sensitivity, which are guided by previous literature (19–21). More specifically, force-time metrics from both the braking and propulsive phase were chosen, reflecting both strategy (e.g., braking phase duration), and outcome or kinetic characteristics (e.g., braking net impulse, jump height). Further, metrics that have been suggested to be sensitive to neuromuscular fatigue in previous literature were included in the analysis (19, 20). Intra- and inter-day reliability statistics were calculated for all metrics of interest across the 3-week baseline period, with specific calculations highlighted in the statistical analysis section. Selected force-time metrics and definitions may be found in Table 1.

**Table 1:** Dependent variables examined in the present study and their definitions and abbreviations.

External load metrics (Unit)	Abbreviation	Description
Accumulated acceleration load (AU)	AAL	Summary of all load/movements in *x*-, *y*-, and *z*-axes.
Acceleration load medium (AU)	AL_M_	Acceleration load sustained between 2.2 and 3.6 m/s^2^
Acceleration load high (AU)	AL_H_	Acceleration load sustained between 3.6 and 5.0 m/s^2^
Acceleration load very high (AU)	AL_VH_	Acceleration load sustained above 5.0 m/s^2^
Distance at anaerobic Activity (m)	AA_D_	Distance covered while sustaining acceleration load above 4.0 m/s^2^ threshold.
Vertical jump metrics (Unit)		
Body mass (kg)	BW	System mass gathered during weighing phase prior to jump.
Jump height (cm)	JH	Maximal jump height via impulse—momentum calculation.
Jump momentum (m/s*kg)	JM	Vertical center of mass take-off velocity multiplied with athlete body weight.
mRSI (ratio)	mRSI	Jump height divided by time-to-takeoff.
Braking rate of force development (N/s)	BRFD	Average change in force over time during the braking phase.
Braking phase duration (s)	BPD	Total duration of braking phase.
Braking net impulse (N*s)	BNI	Net vertical impulse during the braking phase.
Average braking velocity (m/s)	ABV	Average center of mass velocity during the braking phase.
Countermovement depth (cm)	CMD	Lowest center of mass displacement, transition from braking to propulsive phase.
Propulsive phase duration (s)	PPD	Total duration of propulsive phase.
Time-to-takeoff (s)	TTT	Duration from start of the countermovement until take-off.
10-5 Hop test metric (Unit)		
Top 3 jumps average jump height (cm)	T3 JH	Average of highest three jump heights.
Top 3 jumps average mRSI (ratio)	T3 mRSI_A_	Average mRSI^a^ of highest three jump heights.
Top 3 jumps peak mRSI (ratio)	T3 mRSI_P_	Peak mRSI^a^ of highest three jump heights.

AU, arbitrary unit; CMJ, countermovement vertical jump.

^a^ mRSI = jump height calculated using time in the air divided by the total time taken from initial contact to the instant of take-off.

### 2.4 10-5 hop testing

Procedures to collect 10-5 hop test data as a test reflecting the use of a fast SSC were adapted from previous studies (30, 31). On test days athletes performed two 10-5 hop test trials with their hands placed on their hips, with individual trials being separated by 15–30 s. Technology used and procedures done prior to the start of each trial are identical to the CMJ procedures. Athletes were instructed to jump as high as possible on each individual hop, while spending as little time as possible on the ground in-between hops. For each trial, the top three jumps were used to calculate average jump height, average reactive strength index modified (mRSI), and peak mRSI. Selected force-time metrics and definitions may also be found in Table 1. In line with the CMJ, intra-, and inter-day reliability statistics were calculated for all metrics of interest across the 3-week baseline period.

### 2.5 External load data collection

Methodologies used to quantify external load data were adapted from previous literature (32). Athletes wore an inertial measurement unit (IMU; KINEXON Precision Technologies, Version 1.0, Munich, Germany), sampling at 20 Hz during every official practice during baseline week 3, as well as during the fatiguing 5-day high-intensity training phase. In line with manufacturer guidelines, IMU units were placed in a pouch that was clipped to the back of each athletes' shorts, located near the posterior superior iliac spine, above the right posterior hip pocket. The location of the IMU units remained consistent over the study duration. Session recordings during practices and games were started and ended at the same time for each athlete on the team (32). Several external load metrics were derived to conceptualize the difference in the weekly load sum during baseline week 3, compared to the fatiguing 5-day high-intensity training phase. These metrics were calculated based on proprietary algorithms, and captured all movements in the *x*, *y*, and *z*-axes, quantifying load sustained from motion, including jumps, and impacts for instance. Further metric names and definitions may be found in Table 1. Thresholds/zones for acceleration-based load metrics were adapted from earlier research, and in some cases slightly modified, in line with recommendations by the team's strength and conditioning staff (33, 34, 35).

### 2.6 Statistical analyses

Both external load data, as well as CMJ and 10-5 hop force-time metrics were downloaded from each of the company's (KINEXON Precision Technologies, Version 1.0, Munich, Germany and Hawkin Dynamics, Westbrook, ME, USA) highly secured, central, cloud-based locations, and entered into an excel spreadsheet (Microsoft, Redmon, WA, USA), prior to importing the excel file to RStudio (Version 1.4.1106), where further data treatment and statistical analyses were performed. Based on the three-week baseline period, inter-, and intraday ICCs were calculated, and interpreted according to previous literature (36). Further, inter-day SEMs and MDs were calculated in line with suggestions by Weir (37). While the ICC is unitless, the SEM provides an absolute index of reliability and has the same units as the metric of interest (37). Additionally, the MD was calculated by constructing a 90% confidence interval around the SEM and may be used to in our case identify whether or not a change from baseline is to be identified as real (37). This method presents as more conservative with regards to detecting performance changes, compared to other statistical approaches such as comparisons to the coefficient of variation, or the smallest worthwhile change.

For external load metrics, the sum of baseline week three, as well as for the fatiguing 5-day high-intensity training phase were calculated, and compared using a paired samples *t*-test, with Cohen's *d* effect sizes used to quantify the magnitude of difference between weeks. Cohen's *d* effect sizes were interpreted in line with previous suggestions (38), and differences were further visualized using Gardner-Altman Plots (39). The Gardner-Altman plots were generated in RStudio using the “dabestr” package.

To study the neuromuscular fatigue response to the fatiguing training phase, mixed effect models were fit with the respective force-time metric as the dependent variable, time as the fixed factor, and athlete ID as the random effect intercept. These models were fit using the *lme4* package in RStudio. In case of a significant fixed effect omnibus test, further pairwise comparisons were performed using a Bonferroni correction. Data were checked for homoscedasticity and normal distribution of residuals through Q-Q plots and residuals histograms. Instead of averaging force-time data at respective timepoints, all data points were entered into the model. Random intercept only models were compared to random intercept and slope models using the Bayesian Information Criterion (BIC). In the majority of cases, the intercept only models presented with lower BIC values, suggesting their adoption. Further, the magnitude of change from baseline was identified through calculating standardized effect sizes using previous suggestions for multilevel models (40). These effect sizes were interpreted as highlighted above (40). Lastly, at each post-baseline timepoint, a percentage was calculated of the number of individual athletes that dropped below the MD threshold for each metric. Statistical inferences were made using an alpha level of *p* ≤ 0.05.

## 3 Results

**Figure 2:**
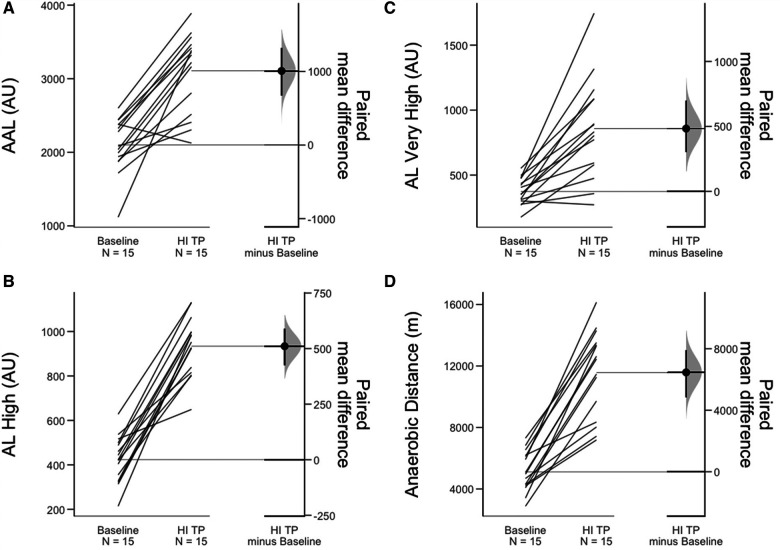
Gardner-Altman plots displaying paired mean differences and associated confidence intervals for the four significant external load metrics between baseline week 3 and the fatiguing high-intensity training period. The significant external load metrics were (**A**) Accumulated acceleration load (AU), (**B**) Acceleration load high (AU), (**C**) Acceleration load very high (AU), and (**D**) Anaerobic distance (m). *Note: HI TP, High Intensity Training Period; AU, arbitrary unit; AAL, accumulated acceleration load; AL, acceleration load.

**Figure 3:**
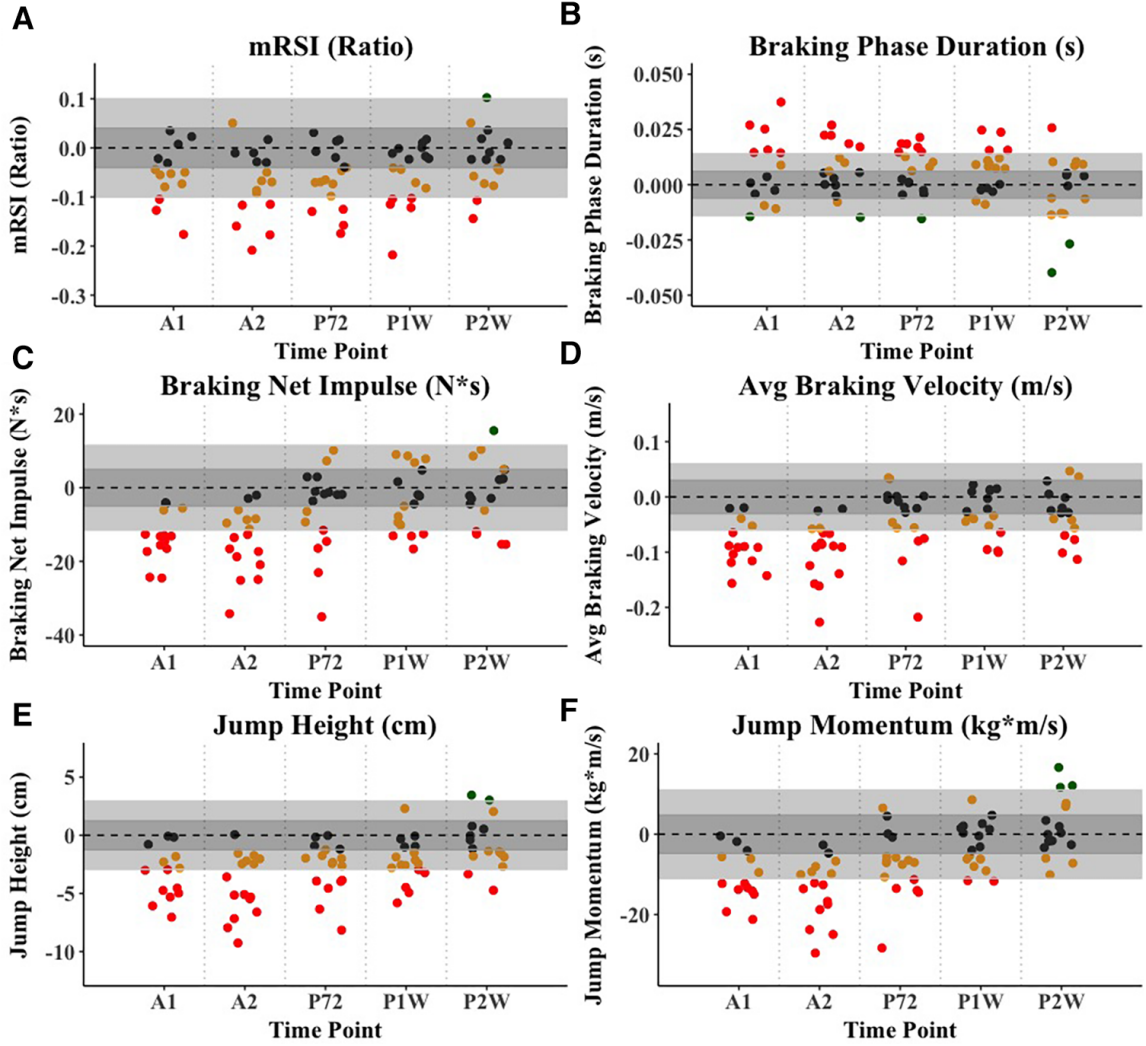
Example visualization of fatigue sensitivity data for selected CMJ-derived force-time metrics. (**A**) mRSI (Ratio), (**B**) Braking phase duration (s), (**C**) Braking net impulse (N*s), (**D**) Average braking velocity (m/s), (**E**) Jump height (cm), (**F**) Jump momentum (kg*m/s). The light grey area displays the MD, with individual data points representing individual athletes, while the dark grey area displays the SEM. Athletes are colored red if they exceed the MD threshold, suggesting a negative performance adaption, orange if they exceed the SEM threshold but not the MD, and black if they do not exceed the SEM threshold. Lastly, athletes are colored green if they exceed the MD threshold, suggesting a positive adaption. *Note: A1, Acute 1; A2, Acute 2; P72, Post 72 hours; P1W, Post one week; P2W, Post two weeks.

**Figure 4:**
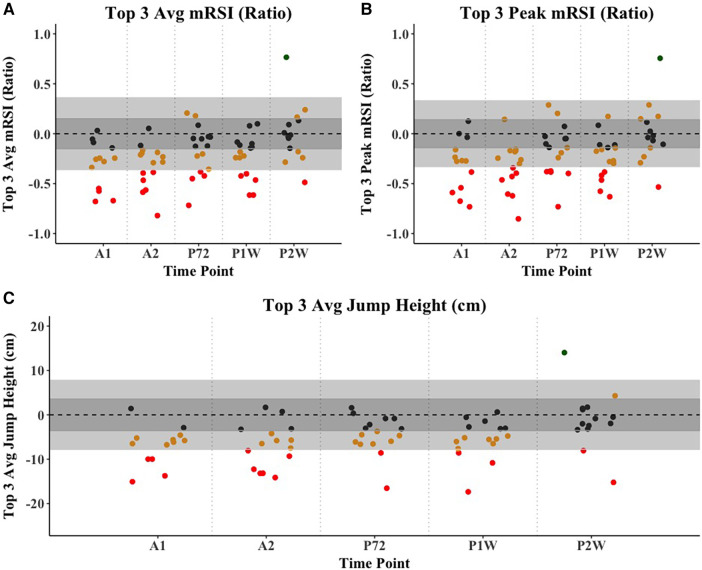
Example visualization of fatigue sensitivity data for the 10-5 hop test-derived force-time metrics, which were (**A**) Average mRSI from the top 3 jumps (Ratio), (**B**) Peak mRSI from the top 3 jumps (Ratio), and (**C**) Average jump height from the top 3 jumps (cm). The light grey area displays the MD, with individual data points representing individual athletes, while the dark grey area displays the SEM. Athletes are colored red if they exceed the MD threshold, suggesting a negative performance adaption, orange if they exceed the SEM threshold but not the MD, and black if they do not exceed the SEM threshold. Lastly, athletes are colored green if they exceed the MD threshold, suggesting a positive adaption. *Note: A1, Acute 1; A2, Acute 2; P72, Post 72 hours; P1W, Post one week; P2W, Post two weeks.

Intra-, and inter-day ICCs (not to be confused with the random component ICCs from the mixed effects model) for all respective metrics of interest ranged from 0.66 to 0.98 and may be found in Table 2. External load comparisons between the sum of baseline week three and the sum of the fatiguing 5-day high-intensity training phase suggest significantly greater AAL (ES = 2.2), AL_H_ (ES = 4.3), AL_VH_ (ES = 1.7), as well as AA_D_ (ES = 2.6). AL_M_ was not found to be different between the two training weeks. Table 3 and Figure 2 display training load comparison results. For the mixed effects model, the following CMJ force-time metrics displayed significant fixed effects omnibus tests: Jump height (*F* = 41.1, Random component ICC (ICC_R_) = 0.82), body mass (*F* = 22.4, ICC_R_ = 0.99), Jump momentum (*F* = 44.1, ICC_R_ = 0.95), countermovement depth (*F* = 10.4, ICC_R_ = 0.80), braking RFD (*F* = 5.4, ICC_R_ = 0.83), braking Phase duration (*F* = 8.2, ICC_R_ = 0.83), Propulsive phase duration (*F* = 3.1, ICC_R_ = 0.90), braking Net impulse (*F* = 25.2, ICC_R_ = 0.76), and average braking velocity (*F* = 26.8, ICC_R_ = 0.60), mRSI (*F* = 16.7, ICC_R_ = 0.80). Further, the following metrics derived from the 10-5 hop test displayed significant fixed effect omnibus test: Top 3 Jumps average jump height (*F* = 28.4, ICC_R_ = 0.74), Top 3 jumps average mRSI (*F* = 23.6, ICC_R_ = 0.76), Top 3 Jumps peak mRSI (*F* = 23.4, ICC_R_ = 0.76). Table 4 displays descriptive statistics presented as means and standard errors, while Table 5 presents fixed-effects parameter estimates derived from the mixed effects model. Example fatigue sensitivity data is visualized in Figures 3, 4.

**Table 2:** Baseline data for metrics of interest from the CMJ and 10-5 hop test.

Metric	Test	SEM	MD	ICC	ICC_Low_	ICC_High_
Intra-day reliability						
JH (cm)	CMJ	1.32	3.06	0.94	0.87	0.98
JM (kg*m/s)	CMJ	4.05	9.4	0.98	0.97	0.99
mRSI (Ratio)	CMJ	0.05	0.12	0.91	0.76	0.96
BRFD (N/s)	CMJ	3,986	9,244	0.77	0.63	0.90
BPD (s)	CMJ	0.01	0.02	0.88	0.75	0.96
BNI (N*s)	CMJ	7.51	17.4	0.91	0.80	0.96
ABV (m/s)	CMJ	0.05	0.12	0.83	0.64	0.93
CMD (cm)	CMJ	2.11	4.88	0.87	0.61	0.96
PPD (s)	CMJ	0.01	0.02	0.95	0.89	0.98
TTT (s)	CMJ	0.04	0.09	0.85	0.68	0.94
T3 JH (cm)	10-5	1.91	4.43	0.93	0.79	0.98
T3 mRSI_A_ (Ratio)	10-5	0.10	0.23	0.94	0.78	0.98
T3 mRSI_P_ (Ratio)	10-5	0.11	0.26	0.91	0.73	0.98
Inter-day Reliability						
JH (cm)	CMJ	1.24	2.93	0.95	0.88	0.98
JM (kg*m/s)	CMJ	4.74	11.0	0.98	0.95	0.99
mRSI (Ratio)	CMJ	0.04	0.10	0.90	0.76	0.97
BRFD (N/s)	CMJ	2,001	4,640	0.96	0.90	0.99
BPD (s)	CMJ	0.006	0.014	0.97	0.92	0.99
BNI (N*s)	CMJ	4.96	11.5	0.95	0.86	0.98
ABV (m/s)	CMJ	0.03	0.06	0.92	0.81	0.98
CMD (cm)	CMJ	1.47	3.40	0.94	0.84	0.98
PPD (s)	CMJ	0.008	0.02	0.97	0.91	0.99
TTT (s)	CMJ	0.04	0.10	0.86	0.68	0.95
T3 JH (cm)	10-5	3.35	7.78	0.66	0.25	0.91
T3 mRSI_A_ (Ratio)	10-5	0.15	0.36	0.83	0.56	0.94
T3 mRSI_P_ (Ratio)	10-5	0.14	0.33	0.87	0.66	0.95

JH, jump height; JM, jump momentum; mRSI, modified reactive strength index; BRFD, braking rate of force development; BPD, braking phase duration; BNI, braking net impulse; ABV, average braking velocity; CMJ, countermovement depth; PPD, propulsive phase duration; TTT, time-to-takeoff; T3 JH, top 3 jumps jump height; T3 mRSI_A_, top 3 jumps average modified reactive strength index; T3 mRSI_P_, top 3 jumps peak modified reactive strength index.

**Table 3:** External load comparison of baseline week three vs. the fatiguing 5-day high-intensity training period.

Metric	Sum baseline Week 3	Sum fatiguing 5-day high-intensity period	ES (CI_90_)
	Mean ± SD	Mean ± SD	
AAL (AU)*	2,099 ± 375	3,106 ± 538	1.77 (1.06 to 2.44)
AL_M_ (AU)	428 ± 106	479 ± 121	0.22 (−0.11 to 0.76)
AL_H_ (AU)*	423 ± 105	934 ± 133	3.16 (2.07 to 4.19)
AL_VH_ (AU)*	375 ± 103	858 ± 387	1.44 (0.81 to 2.04)
AA_D_ (m)*	5,120 ± 1,308	11,581 ± 2,825	2.61 (1.68 to 3.50)

AU, arbitrary unit; AAL, accumulated acceleration load; AL_M_, acceleration load medium; AL_H_, acceleration load high; AL_VH_, acceleration load very high; AA_D_, anaerobic activity distance; ES, effect size; CI_90_, 90% confidence interval.

“*” = significantly larger than baseline week 3.

**Table 4:** Descriptive data on respective timepoints for CMJ and 10-5 hop test metrics. Data is presented as means and standard errors.

Metric	Test	Baseline	Acute1	Acute2	Post 72h	Post 1 Week	Post 2 Weeks
BW (kg)	CMJ	92.2 ± 2.81	92.1 ± 2.82	92.1 ± 2.82	92.8 ± 2.82*	93.8 ± 2.82*	93.6 ± 2.82*
JH (cm)	CMJ	43.4 ± 1.12	40.1 ± 1.2*	39.2 ± 1.2*	40.4 ± 1.1*	41.1 ± 1.1*	42.6 ± 1.2
JM (kg*m/s)	CMJ	268 ± 7.1	257 ± 7.1*	254 ± 7.1*	260 ± 7.1*	265 ± 7.1	269 ± 7.1
mRSI (ratio)	CMJ	0.66 ± 0.03	0.61 ± 0.03*	0.59 ± 0.03*	0.60 ± 0.03*	0.61 ± 0.03*	0.64 ± 0.03
BRFD (N/s)	CMJ	13,006 ± 1,913	11,425 ± 1,976*	10,316 ± 1,913	11,766 ± 1,952	11,371 ± 1,957	12,994 ± 1,964
BPD (s)	CMJ	0.143 ± 0.01	0.151 ± 0.01*	0.151 ± 0.01*	0.150 ± 0.01*	0.150 ± 0.01*	0.139 ± 0.01
BNI (N*s)	CMJ	131 ± 4.13	117 ± 4.33*	116 ± 4.30*	124 ± 4.26*	128 ± 4.28	130 ± 4.30
ABV (m/s)	CMJ	0.92 ± 0.02	0.83 ± 0.02*	0.82 ± 0.02*	0.87 ± 0.02*	0.88 ± 0.02*	0.89 ± 0.02*
CMD (cm)	CMJ	29.8 ± 1.11	28.1 ± 1.2*	27.3 ± 1.2*	28.9 ± 1.1	29.5 ± 1.1	28.7 ± 1.1
PPD (s)	CMJ	0.23 ± 0.01	0.23 ± 0.01	0.23 ± 0.01	0.23 ± 0.01	0.24 ± 0.01	0.23 ± 0.01
TTT	CMJ	0.68 ± 0.02	0.69 ± 0.02	0.68 ± 0.02	0.70 ± 0.02	0.70 ± 0.02	0.68 ± 0.02
T3 JH (cm)	10-5	31.1 ± 1.52	24.2 ± 1.63*	24.0 ± 1.61*	26.6 ± 1.60*	25.8 ± 1.60*	29.8 ± 1.62
T3 mRSI_A_ (ratio)	10-5	1.40 ± 0.08	1.08 ± 0.08*	1.06 ± 0.08*	1.23 ± 0.08*	1.17 ± 0.08*	1.37 ± 0.08
T3 mRSI_P_ (ratio)	10-5	1.47 ± 0.08	1.15 ± 0.09*	1.12 ± 0.09*	1.30 ± 0.09*	1.23 ± 0.09*	1.43 ± 0.09

JH, jump height; JM, jump momentum; mRSI, modified reactive strength index; BRFD, braking rate of force development; BPD, braking phase duration; BNI, braking net impulse; ABV, average braking velocity; CMJ, Countermovement depth; PPD, propulsive phase duration; TTT, time-to-takeoff; T3 JH, top 3 jumps jump height; T3 mRSI_A_, top 3 jumps average modified reactive strength index; T3 mRSI_P_, top 3 jumps peak modified reactive strength index.

“*” and bold text = significantly different from baseline.

**Table 5:** Mixed effects model parameter estimates and fatigue sensitivity data.

Metric	Acute 1			Acute 2		
	Estimate (CI_90_)	ES	% Below MD	Estimate (CI_90_)	ES	% Below MD
BW (kg)	−0.01 (−0.37 to 0.35)	0	7%	−0.06 (−0.41 to 0.28)	0.01	13%
JH (cm)	−3.30 (−3.90 to −2.67)	0.64	57%	−4.24 (−4.88 to −3.66)	0.82	56%
JM (kg*m/s)	−10.43 (−12.43 to −8.42)	0.33	57%	−13.39 (−15.30 to −11.48)	0.43	56%
mRSI (Ratio)	−0.06 (−0.07 to −0.04)	0.41	21%	−0.07 (−0.09 to −0.05)	0.53	31%
BRFD (N/s)	−1,573 (−2,625 to −522)	0.18	7%	−2,683 (−3,687 to −1,680)	0.30	6%
BPD (s)	0.008 (0.004 to 0.011)	0.24	43%	0.008 (0.004 to 0.011)	0.24	31%
BNI (N*s)	−6.65 (−10.40 to −2.90)	0.67	71%	−8.21 (−11.79 to −4.63)	0.74	56%
ABV (m/s)	−0.09 (−0.10 to −0.07)	0.93	71%	−0.10 (−0.11 to −0.08)	1.04	75%
CMD (cm)	−0.71 (−0.97 to −0.44)	0.34	15%	−1.0 (−1.25 to −0.74)	0.48	40%
PPD (s)	0.0006 (−0.004 to 0.003)	0.01	7%	0.005 (−0.008 to −0.001)	0.11	0%
TTT (s)	0.013 (−0.003 to 0.03)	0.12	0%	0.002 (−0.013 to 0.017)	0.02	0%
T3 JH (cm)	−7.01 (−8.36 to −5.66)	0.98	31%	−7.16 (−8.46 to −5.89)	1.01	40%
T3 mRSI_A_ (Ratio)	−0.31 (−0.38 to −0.25)	0.83	31%	−0.33 (−0.40 to −0.27)	0.89	40%
T3 mRSI_P_ (Ratio)	−0.32 (−0.39 to −0.25)	0.80	38%	−0.35 (−0.41 to −0.28)	0.88	47%
	Post 72h			Post 1 Week		
BW (kg)	0.68 (0.35 to 1.01)	0.06	0%	1.69 (1.36 to 2.02)	0.14	0%
JH (cm)	−3.07 (−3.63 to −2.49)	0.59	35%	−2.29 (−2.87 to −1.70)	0.44	29%
JM (kg*m/s)	−7.98 (−9.79 to −6.17)	0.26	29%	−2.42 (−4.28 to −0.57)	0.08	12%
mRSI (Ratio)	−0.07 (−0.08 to −0.05)	0.47	24%	−0.06 (−0.07 to −0.04)	0.41	29%
BRFD (N/s)	−1,232 (−2,183 to −282)	0.14	0%	−1,627 (−2,601 to −655)	0.18	6%
BPD (s)	0.007 (0.004 to 0.010)	0.21	35%	0.007 (0.003 to 0.010)	0.20	25%
BNI (N*s)	−6.81 (−9.36 to −4.36)	0.34	29%	−3.10 (−5.71 to −0.49)	0.16	24%
ABV (m/s)	−0.043 −(0.059 to −0.028)	0.47	24%	−0.031 (−0.047 to −0.015)	0.33	24%
CMD (cm)	−0.94 (−1.55 to −0.33)	0.18	6%	−0.36 (−0.99 to 0.28)	0.07	0%
PPD (s)	−0.0005 (−0.004 to 0.002)	0.01	0%	0.002 (−0.002 to 0.005)	0.05	0%
TTT (s)	0.02 (0.007 to 0.04)	0.19	6%	0.02 (0.006 to 0.04)	0.19	6%
T3 JH (cm)	−4.26 (−5.87 to −3.35)	0.65	13%	−5.36 (−6.63 to −4.11)	0.76	19%
T3 mRSI_A_ (Ratio)	−0.17 (−0.23 to −0.10)	0.44	25%	−0.23 (−0.29 to −0.16)	0.60	31%
T3 mRSI_P_ (Ratio)	−0.17 (−0.23 to −0.10)	0.43	31%	−0.24 (−0.30 to −0.17)	0.60	31%
	Post 2 Weeks					
BW (kg)	1.44 (1.10 to 1.79)	0.12	0%			
JH (cm)	−0.81 (−1.40 to −0.20)	0.15	13%			
JM (kg*m/s)	1.64 (−0.28 to 3.55)	0.05	0%			
mRSI (Ratio)	−0.02 (−0.04 to −0.01)	0.17	13%			
BRFD (N/s)	−4.43 (−1,010 to 1,001)	0	6%			
BPD (s)	−0.004 (−0.008 to −0.001)	0.13	6%			
BNI (N*s)	−0.98 (−3.68 to −1.72)	0.05	25%			
ABV (m/s)	−0.028 (−0.044 to −0.011)	0.30	25%			
CMD (cm)	−1.12 (−1.78 to −0.48)	0.22	14%			
PPD (s)	−0.006 (−0.010 to −0.003)	0.15	0%			
TTT (s)	0.008 (−0.01 to 0.02)	0.08	6%			
T3 JH (cm)	−1.35 (−2.67 to 0.02)	0.19	14%			
T3 mRSI_A_ (Ratio)	−0.03 (−0.10 to 0.04)	0.08	7%			
T3 mRSI_P_ (Ratio)	−0.03 (−0.10 to 0.04)	0.08	7%			

JH, jump height; JM, jump momentum; mRSI, modified reactive strength index; BRFD, braking rate of force development; BPD, braking phase duration; BNI, braking net impulse; ABV, average braking velocity; CMJ, countermovement depth; PPD, propulsive phase duration; TTT, time-to-takeoff; T3 JH, top 3 jumps jump height; T3 mRSI_A_, top 3 jumps average modified reactive strength index; T3 mRSI_P_, top 3 jumps peak modified reactive strength index; ES, effect size; CI_90_, 90% confidence interval.

## 4 Discussion

This study aimed to investigate the SSC fatigue response and sensitivity of selected force-time metrics from a fast and a slow SSC movement, to a 1-week high intensity fatiguing phase of training in NCAA Division-I basketball players. Primary findings suggested that slow and fast SSC task-related force-time metrics are sensitive to a stressful phase of training. Over a total study duration of six weeks, athletes performed baseline testing, two test sessions during the fatiguing phase of training (6 h post completion), as well as testing at 72 h post, one week post, and two weeks post. When compared to the weekly training sum at baseline, during the one-week high intensity training phase, athletes were exposed to very large increases in the weekly sum for selected external load metrics (ES = 1.44–3.16), suggesting that the athletes experienced fatigue acutely, as well as potential longer lasting reductions in performance.

The reliability data gathered over the three-week baseline period showed moderate to excellent intra- (ICC = 0.77–0.98), and inter-day (ICC = 0.66–0.98) reliability for metrics from both tasks, which suggests that similar to previous research, SSC function during and after a fatiguing phase of training may be sensitively detected (18). More specifically, a number of metrics showed significant reductions from baseline, across different time-points, with effect sizes and the number of athletes falling below the MD threshold varying.

At time points A1 and A2, which were performed two days and four days into the fatiguing training phase (6 h post training completion), JH, BNI, ABV, as well as T3 JH, T3 mRSI_A_, and T3 mRSI_P_ showed the largest reductions in performance when compared to baseline. When looking at the percentage of athletes that experienced performance reductions greater than the MD threshold, ABV seemed most sensitive, with 71%, and 75% of athletes falling below the MD threshold, respectively. Interestingly, and likely influenced by a reduction in CMD, TTT remained unchanged, which has previously been suggested to display good sensitivity to neuromuscular fatigue (20, 21). This reduction in CMD likely also helps explain the moderate to large reductions in JH, given that athletes had less time and range of motion to attain sufficient center of mass velocity (25). While labeled as acute within our study, both A1 and A2 were performed approximately 6 h following the completion of the training session on their respective days. In line with the previously highlighted bimodal recovery concept (9), performance decreases at A1 and A2 may be explained by muscle soreness, rather than metabolic fatigue. There is little research that has looked at a fatigue-response in that specific timeframe. Yoshida et al. recently showed that at 6 h following basketball-related high-intensity exercises, moderate to large performance decrements in peak force, flight-time:contraction-time, as well as positive impulse, unweighting impulse, eccentric rate of force development and duration, when compared to baseline (41). Metrics such as JH or peak power have been shown to be sensitive to fatigue or sport-specific training exposure at timepoints closer to the commencement of the fatiguing activity (4, 24). Others have reported opposite or conflicting findings when looking at traditional or outcome-based metrics such as JH (4, 42, 43).

Further, in discussing post-fatigue testing, it must be acknowledged that P72h was performed following two days of rest, while P1W, and P2W were performed at the ends of normal training weeks, consisting of basketball-specific practices and regular strength and conditioning sessions, rather than complete rest, or high-intensity fatiguing training. At P72h, while a general trend back toward baseline was observed, JH, ABV, as well as T3 JH and mRSI showed the greatest reductions from performance (ES = 0.47–0.59). This decrease in mRSI in our data is mostly explained by the reductions in JH (ES = 0.59), rather than TTT (ES = 0.19), suggesting that athletes still maintained a similar jump strategy, despite being seemingly fatigued. With regards to the number of athletes that dropped below the MD threshold, 35% of athletes still displayed JH reductions greater than the MD. Interestingly, while only showing a small ES (0.21), 35% of all athletes also displayed BPD increases greater than the respective MD threshold. This suggests that while still exhibiting depressed center of mass range of motion, some athletes presented with a jump strategy suggesting an elongated braking phase. Moreover, while it is important to factor in the magnitude of change, BPD showed statistically significant increases at all time-points besides P2W. This same trend was observed for mRSI, JH, as well as all three 10-5 hop test metrics. In the study by Gathercole et al., at 72 h post fatigue protocol, time-based metrics such as PPD, TTT, and BPD all showed small but significantly greater values compared to baseline, which authors attribute to a shift in neuromuscular strategy during the CMJ (20). In our results, this suggestion was only reflected in BPD, with TTT and PPD remaining unchanged. Furthermore, Gathercole et al. highlighted a significant, small to moderate (ES = 0.44) depression in flight-time:contraction-time (i.e., mRSI), which closely aligns with our findings (20). Similarly, Heishman et al. suggested moderate decreases in mRSI, which were paralleled with increases in external training load over an in-season period in collegiate basketball players (34).

When looking at the more chronic adaptions to the fatiguing high-intensity training phase, it is observable that at P1W most CMJ metrics continued to trend back toward baseline, with JH, mRSI, BPD, BNI, and ABV displaying small to moderate effect sizes, with 25%–29% of athletes still below the MD threshold for said metrics. On the other hand, in analyzing metrics from the 10-5 hop test, a secondary reduction was observed, with moderate to large decreases in performance for T3 JH, and both T3 mRSI_A_, and T3 mRSI_P_. However, T3 JH presented with only a moderate inter-day ICC, accompanied by a fairly wide confidence interval, which must be considered in interpreting these data. While on a different timescale, and largely speculative, this finding, and the overall performance trend over time for 10-5 hop test metrics aligns with the bimodal recovery construct proposed in previous literature (9). Earlier research has described a frequent SSC fatigue-induced loss of tolerance to ground impact, which may be reflected in our 10-5 hop test findings (44–46). During our maximal, reactive hops, it is possible that reductions in performance were impacted by reductions in soleus muscle pre-activation, leading to decreased EMG activity and force output during the stretch-reflex phase, which has been suggested as an explanation for fatigue-induced performance decrements in maximal, repeated drop jumps (46, 47). These findings have been proposed to occur primarily in the delayed phase of recovery, explaining why moderate to large reductions in hop test performance were still present at P1W (47). While speculative, this inadequate neural drive may be viewed as an attempt of the neuromuscular system to protect the muscle-tendon unit from further stress or even damage and could suggest the development of central fatigue, which tends to set in later than metabolic or peripheral fatigue (1, 9, 48). Furthermore, across most time-points, metrics derived from the 10-5 hop test displayed larger effect sizes compared to metrics derived from the CMJ. This partially aligns with earlier work demonstrating acute performance impairments for high ground-impact SSC tasks (e.g., drop-, or repeated jumps), with no significant acute impairments in low ground-impact SSC tasks (e.g., CMJ), or non-SSC tasks (e.g., squat jump) (47, 49).

At P2W, most metrics have mostly returned to baseline. However, ABV still displayed a small (ES = 0.30) but significant reduction from baseline values, with 25% of athletes still below the rather conservative MD threshold. Similarly, while not significant, and only showing a trivial effect size, for BNI 25% of athletes maintained below the MD threshold, despite athletes possessing significantly greater body weight values, compared to baseline. The previous findings further agree with previously mentioned hypotheses about neural adjustments in the later recovery phases post-fatigue, aiming to protect the muscle-tendon unit from damage. More specifically, the previously observed reduction in reflex sensitivity, reflected in reductions in pre-activation of the soleus muscle, as well as reduced EMG activity and performance during the braking phase may help explain the reduced ABV and BNI (43, 44). It seems the eccentric phase of the CMJ may be particularly sensitive to SSC fatigue at acute, as well as more chronic timepoints, which is in agreement with limited previous research findings (20, 41, 50). In our data, BRFD may not have displayed significant, longer lasting performance reductions, due to one athlete consistently displaying a movement strategy lending itself to the generation of larger rates of force development across the braking phase of the jump, when compared to the rest of the team. Particular attention with regards to recovery and training modifications may be given to athletes below the MD threshold at later time-points (i.e., Post 1 Week and Post 2 Weeks).

Recent studies have emphasized the need to quantify and interpret an athlete's jump strategy, hypothesizing that in fatigued states, skilled jumpers likely adjust their movement strategy in order to avert a decreased performance output (19, 20, 22). In a recent commentary on the selection process of CMJ variables, Bishop et al. highlighted TTT and PPD to be two particularly sensitive metrics (19). Interestingly, in our study, these two metrics remained mostly unchanged across all acute and post-fatigue time-points (19). Therefore, in our results, changes in mRSI were largely driven by decreases in jump height, which is in disagreement with previous literature, and emphasizes the need to interpret the numerator and denominator when analyzing ratio metrics (19, 20, 22). Similarly, within the 10-5 hop test, reductions in mRSI were largely driven by decreases in JH, with athletes maintaining average ground-contact durations. For the CMJ, our results suggest that strategy-based metrics, primarily from the eccentric phase (e.g., ABV, BPD) were altered acutely and chronically. Furthermore, displacement of the center of mass (i.e., CMD) was primarily diminished acutely, likely influenced by factors related to tissue disruption, rather than metabolic fatigue. Previous literature has suggested decreased angular displacement of the knee joint during a vertical jump following fatigue to the knee extensor muscles (51).

**Figure 5:**
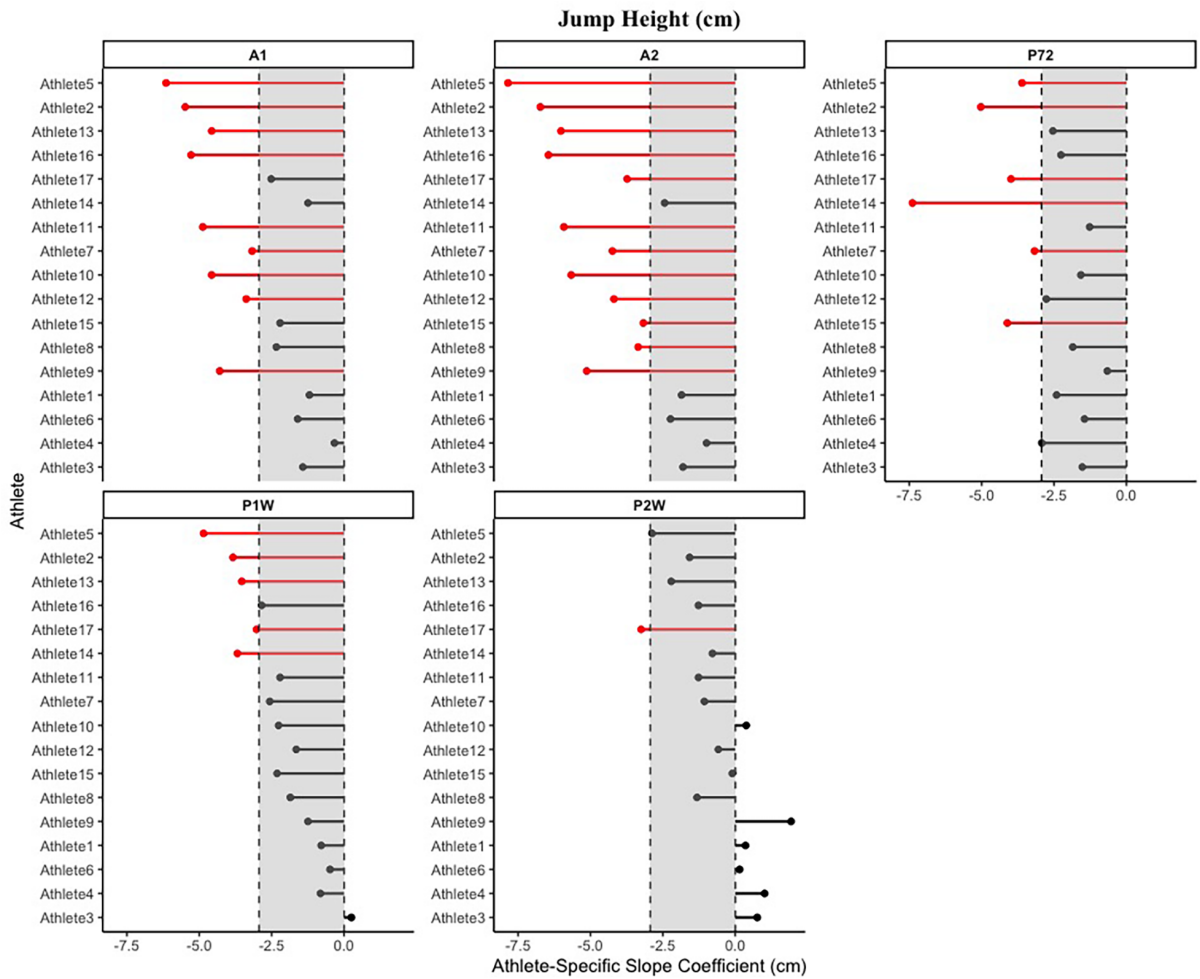
Example visualization of athlete-specific slope coefficients derived from random intercept and slope models that can be used to determine athlete-specific fatigue responses. The shaded grey area displays the MD, with red lines representing athletes going beyond the MD threshold at respective time-points.

Identifying the number of athletes below a certain threshold, such as the MD in our case, allows practitioners to dedicate particular attention to the recovery and adaption to drastically increased levels of high-intensity training exposure for respective athletes. This poses as important, given the previously highlighted individuality of recovery timelines following SSC fatigue (9). It seems likely that the multilevel model approach used in this study was able to capture some of the variation in the data that was athlete specific, which is shown in the greater model performance, when compared to a simple fixed effect model. While in our mixed effect models, the random intercept only variant mostly outperformed the model including both a random slope and intercept, sport scientist's may consider the implementation of the latter model. This allows for the generation of athlete-specific coefficients, which may be used to further individualize the training and recovery timelines. An example of this can be seen in Figure 5, in which athlete-specific slope coefficients derived from a random intercept and slope model are visualized for each time-point post-baseline. The grey shaded area indicated the previously established MD, and athletes are colored red if they exceed this threshold. This allows sport scientists to identify how individual athletes are responding to the heightened training stress, allowing for the modification of training or recovery parameters.

Authors believe this study effectively contributes to the body of literature; however, certain methodological limitations or considerations should still be acknowledged. Firstly, it is of importance to consider the post-fatigue timeline. As previously mentioned, P72h testing was performed following 2 days of rest, while P1W and P2W testing were performed at the end of each normal training week post-fatigue, respectively. However, the two weeks following the fatiguing protocol were also the first two weeks of the NCAA 20-h period, suggesting an increased volume in basketball-specific activity. Therefore, true recovery with no physical activity was not achievable. This in combination with the length of our fatiguing training phase likely explains the delay in recovery, reflected in our results, when compared to similar studies (20, 41). Regardless, our data suggested varying recovery timelines, with large acute performance reductions, and in some cases, delayed returns toward baseline. While sufficient education about different factors influencing recovery were provided to the athletes, given the applied and real-world nature of our methods and data, researchers were unable to control for within-athlete factors such as sleep, hydration, and food-intake. The previous two points may be viewed as a limitation. However, we believe they also strengthen the external validity of our study. Moreover, authors believe the highly trained nature of the sample, in combination with the length of the study duration and number of assessment timepoints, allowing for a deeper insight into the acute and chronic neuromuscular fatigue response positively adds to a body of literature that is of interest to applied sport science practitioners. Considerations for future avenues of research may include the simultaneous quantification of subjective or perceptual wellbeing (e.g., muscle soreness, sleep, willingness to train), to further tease out information about NMF and recovery timelines. Furthermore, in our study, thresholds and zones for acceleration load metrics were generalized across the whole team. Future research may consider individualizing said thresholds and zones, acknowledging between-athlete differences in physical parameters. Readers should use caution when trying to generalize findings from our investigation to other populations such as female athletes, as findings may change. While in this study, the sample size of athletes was sufficient from a statistical standpoint, authors had no influence over the quantity of participants used in this study, given the number of active players on the roster. Therefore, future studies may also aim to apply methods used across other sports, as well as genders, age groups, and teams with larger sample sizes.

In summary, besides highlighting the intra-, and inter-day reliability of a number of CMJ and 10-5 hop test metrics, this study documented the acute and chronic fatigue sensitivity of selected force-time metrics from a slow and a fast SSC movement task. Our data suggest that in the CMJ, both traditional metrics such as JH as an outcome metric, as well as alternative metrics reflecting kinetic outputs and movement strategies (e.g., Braking velocity), were sensitive to the stark increase in high-intensity training exposure the athletes experienced in our study, and that changes in a number of these variables interact with each other. The inclusion of the 10-5 hop test suggested a fatigue-induced loss of tolerance to ground impact reflected by performance reductions in metrics related to jump height and reactive strength qualities. These findings emphasize that when monitoring neuromuscular fatigue, variables and assessments may not be looked at individually, but rather as part of a more global monitoring approach. Further, the individuality of recovery timelines with regards to task- and time-specificity should be considered when interpreting variables of interest.

## Data Availability

The raw data supporting the conclusions of this article will be made available by the authors, without undue reservation.
